# Thermoelectric Seebeck effect in oxide-based resistive switching memory

**DOI:** 10.1038/ncomms5598

**Published:** 2014-08-20

**Authors:** Ming Wang, Chong Bi, Ling Li, Shibing Long, Qi Liu, Hangbing Lv, Nianduan Lu, Pengxiao Sun, Ming Liu

**Affiliations:** 1 Lab of Nanofabrication and Novel Device Integration, Institute of Microelectronics, Chinese Academy of Sciences, Beijing 100029, China; 2 These authors contributed equally to this work

## Abstract

Reversible resistive switching induced by an electric field in oxide-based resistive switching memory shows a promising application in future information storage and processing. It is believed that there are some local conductive filaments formed and ruptured in the resistive switching process. However, as a fundamental question, how electron transports in the formed conductive filament is still under debate due to the difficulty to directly characterize its physical and electrical properties. Here we investigate the intrinsic electronic transport mechanism in such conductive filament by measuring thermoelectric Seebeck effects. We show that the small-polaron hopping model can well describe the electronic transport process for all resistance states, although the corresponding temperature-dependent resistance behaviours are contrary. Moreover, at low resistance states, we observe a clear semiconductor–metal transition around 150 K. These results provide insight in understanding resistive switching process and establish a basic framework for modelling resistive switching behaviour.

## Abstract (web summary)

Oxide-based resistive switching memory is known to depend on the formation and rupture of a conducting filament, although the mechanism behind this remains debated. Here, the authors measure the Seebeck effect to understand the intrinsic electronic transport mechanism in the conducting filament.

## Graphical abstract

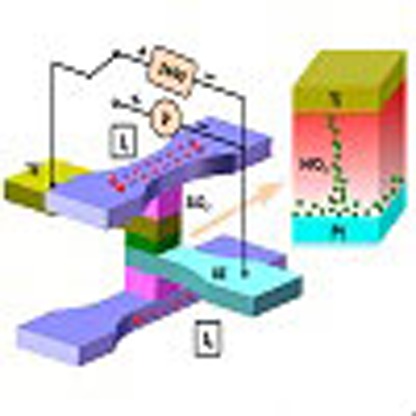

Reversible resistive switching (RS) in the transition metal oxide is a typical electric field- and current-induced phenomenon that the resistance (*R*) strongly relates with the history of applied voltage and current^1^,^2^,^3^. It has attracted considerable attention for future information storage and processing, such as resistive random access memory (RRAM), reconfigurable logic circuit and artificial neuromorphic networks^2^,^3^,^4^,^5^. The switching between high-resistance state and low-resistance state (LRS) usually involves the generation, transport and recombination of defects, such as oxygen vacancies and metal ions, which causes the structure or composition modulation of the ‘active’ switching region, usually called conductive filament (CF)^6^,^7^,^8^,^9^. Understanding the charge transport in the formed CF not only benefits for further understanding RS phenomenon, but also is critical for optimizing RS-based devices in practical applications. Owing to the limitations in spatial resolution and the sensitivity of traditional material and electrical characterization techniques, it is difficult to directly measure the basic electronic transport properties of the formed CF. Although several electronic transport mechanisms have been suggested based on the current–voltage (*I*–*V*) characteristics or temperature dependence of *R*, there are many discrepancies among these proposed electronic transport mechanisms. Even for the similar device structures with the same value of LRS resistance, the proposed transport mechanisms are also quite contradictory^9^,^10^,^11^,^12^,^13^.

Seebeck effect, a typical thermoelectric phenomenon that directly converts the temperature difference to electric field^14^, can provide deeper insights into the energetics of dominant charge transport process, and the Seebeck voltage measurement has become a high sensitive means to image structural or electronic disorder^15^,^16^. The Seebeck coefficient^17^,^18^,


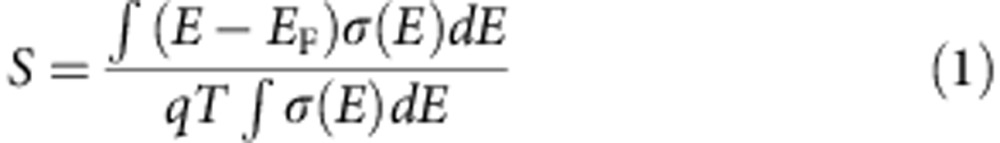


reflects the asymmetry of *σ*(*E*) with respect to *E*_F_, where *σ*(*E*) is the energy-dependent electrical conductivity distribution function, *q* is the basic charge of the carrier, *E*_F_ is the Fermi energy and *T* is the absolute temperature. This asymmetry of *σ*(*E*) can result from energy-dependent carrier scattering, defect energy level around *E*_F_ or the asymmetry of the electronic density of states. Therefore, *S* can reflect the basic charge transport mode, such as the band-like transport or the variable-range hopping transport^18^,^19^. In addition, in contrary to *I*–*V* measurements, the Seebeck voltage does not depend on the number of conductive paths and interfacial contact^14^,^20^ (see Supplementary Fig. 1 and Supplementary Note 1), and thus reveals the intrinsic electronic transport properties.

In the present study, we choose the Ti/HfO_*x*_/Pt RS structure as a typical example to investigate the electronic transport mechanism in metal oxide-based RRAM by measuring the Seebeck effect. The experimental results show that the measured *S* of Ti/HfO_*x*_/Pt sample almost keeps constant at −80 μV K^−1^ when 78 Ω*<R*<10^4^ Ω, and then sharply decreases to about −160 μV K^−1^ for *R*~10^6^ Ω at room temperature. The negative *S* indicates *n*-type electrical transport. The temperature dependence of *S* between 150 and 300 K demonstrates the intrinsic electrical transport mechanism is the same for all different resistance states, although the corresponding *R* versus temperature presents different characteristics. We show that all *S* characteristics under different temperatures and resistances can be well explained by small-polaron hopping model and suggest that the small-polaron hopping process between vacancies dominates the main electron transport in CF. Moreover, a clear semiconductor–metal transition (SMT) is also observed for *R*<~106 Ω when the temperature is below 150 K.

## Results

### Sample configuration and RS characteristics

**Figure 1:**
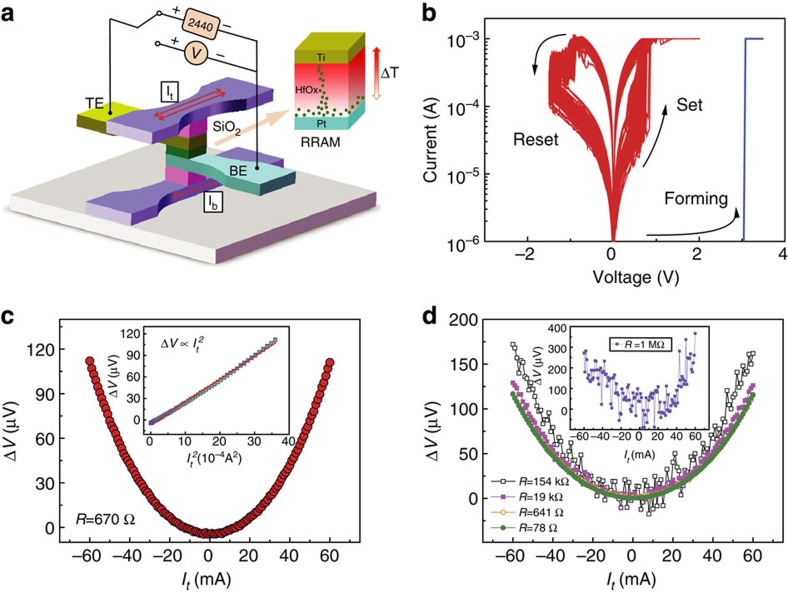
Measurement of the Seebeck voltage in oxide-based RRAM at 300 K. (**a**) Schematic sample structure and corresponding the Seebeck measurement setup. *I*_t_ and *I*_b_ are the heating currents for top and bottom surfaces, respectively. Keithley sourcemeter 2440 is used to operate RRAM and the voltmeter is used to measure the Seebeck voltage. (**b**) *I–V* characteristics of Ti/HfO_*x*_/Pt structure in forming, set and reset process. The initial forming process is done by sweeping voltage from 0 to +4 V with the compliance current *I*_cc_ of 1 mA, and the subsequent reset and set processes are performed by sweeping voltage between −1.5 and 2 V with *I*_cc_ of 100 and 1 mA, respectively. (**c**) Measured Seebeck voltage as a function of *I*_t_ when *I*_b_=0 mA for *R*=670 Ω. The inset shows the linear fitting (solid line) of the Seebeck voltage (symbols) versus *I*_t_^2^. (**d**) Measured Seebeck voltages at several representative resistance states from 78 Ω to 1 MΩ (inset).

The typical W 40/Ti 10/HfO_*x*_ 8/Pt 60/Ti 5 (nm) RRAM structures are used in this experiment. The bottom and top Pt 30/Ti 5 (nm) heating layers and two 70-nm-thick SiO_2_ isolation layers are, respectively, deposited before and after depositing W/Ti/HfO_*x*_/Pt/Ti structure. The schematic sample structure and experimental setup are shown in Fig. 1a, where *I*_b_ and *I*_t_ are used to heat bottom and top surfaces of RRAM structure, and a Keithley sourcemeter 2440 and a voltmeter are used to operate RRAM and measure the Seebeck effect, respectively. The temperature gradient is created by Joule heating bottom and top Pt/Ti layers, and their resistances are used to evaluate the established temperature gradient (see Methods and Supplementary Fig. 2). It should be mentioned that the influence of heating current on RRAM operations can be completely suppressed by two 70-nm SiO_2_ isolation layers. We first preform the usual RRAM measurement^2^, forming, set and reset process, to confirm that our W/Ti/HfO_*x*_/Pt/Ti devices show reliable RS behaviours, as shown in Fig. 1b. After the initial forming process, where voltage is swept from 0 to +4 V with the compliance current *I*_cc_ of 1 mA, the stable hysteresis-like *I*–*V* curves between −1.5 V and 2 V during the subsequent reset and set processes are achieved, indicating that the sample works well as a RRAM.

### Seebeck measurement

The Seebeck coefficient at a given temperature is determined by linearly fitting *ΔV* versus *ΔT* by using *ΔV*=*−SΔT*^21^, where *ΔV* is the measured Seebeck voltage and *ΔT* is the temperature difference across the HfO_*x*_ layer. Before measuring the Seebeck voltage, the sample resistance is first set to a fixed resistance state. Figure 1c shows the typical measured Seebeck voltage as a function of *I*_t_ for *R*=670 Ω when *I*_b_=0 mA at 300 K. It is clearly shown that the measured Seebeck voltage increases with current and does not depend on the current flow direction. The positive Seebeck voltage at the hot end indicates the electrons are the main carriers^14^. The inset of Fig. 1c shows the corresponding voltage as a function of *I*_t_^2^. As *ΔT* is proportional to *I*_t_^2^ (see Supplementary Fig. 2c), the linear dependence of *ΔV* on *I*_t_^2^ indicates that the measured voltage is proportional to the temperature difference at HfO_*x*_ layer, which is the typical Seebeck voltage characteristics. When sweeping *I*_b_ under *I*_t_=0 mA, an identical tendency of measured voltage as a function of applied *I*_b_ is also observed, except that the sign is negative. When *I*_t_=60 mA and *I*_b_=0 mA, *ΔT* is ~1.3 K, which corresponds to an *S*-value of ~84 μV K^−1^ for *R*=670 Ω at 300K. Such large *S*-value of several tens of μV K^−1^, which is much larger than that for typical metal^14^, indicates that the electron transport cannot be understood from the metallic band-like electrical transport model. Figure 1d shows the measured Seebeck voltage as a function of *I*_t_ at several representative resistance states. For the LRS of *R*<10^4^ Ω, the Seebeck coefficient is almost identical for different resistance states; however, for *R*>10^4^ Ω, the value of *S* extracted from linear fitting of *ΔV* versus *ΔT* increases with *R* and can reach up to 160 μV K^−1^ for the *R* of ~10^6^ Ω. For *R*>10^6^ Ω or virgin sample without any RS operation, no Seebeck signal is observed.

**Figure 2:**
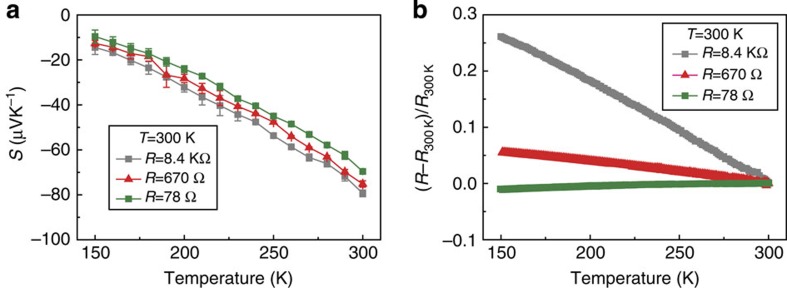
Temperature-dependent measurement results. (**a**) Seebeck coefficient as a function of temperature for several representative resistance states (*R*=78 Ω, 670 Ω and 8.4 kΩ) in oxide-based RRAM. (**b**) Temperature dependence of normalized resistance by the resistance at 300 K (*R*_300 K_).

To offer the further insight into the electronic transport in CF, we measure *S* at different temperatures. Here we still use the sample stage temperature as *T* due to the small *ΔT* compared with sample stage temperature. Figure 2a shows the temperature dependence of *S* between 150 and 300 K at several representative resistance states. The corresponding *R* versus *T* is also simultaneously measured as shown in Fig. 2b. For *R*=8.4 kΩ, *R* decreases with increasing *T*, which corresponds to a conventional semiconductor behaviour, whereas for LRS of *R*=78 Ω, *R* increases with increasing T, which is the typical metallic behaviour. Surprisingly, as shown in Fig. 2a, *S* for all the resistance states show the identical tendency of temperature dependence. As we mentioned above, the Seebeck voltage does not depend on the interfacial contact and only reflects the basic electronic properties of CFs; thus, the identical tendency of *S* versus *T* indicates all the resistance states have the same intrinsic electrical transport mechanism.

As we mentioned above, because the Seebeck voltage does not depend on the interfacial contact and the electrical measurement can be strongly influenced by contact, especially for the LRS, it is reasonable to assume that the different *R* versus *T* tendencies at different resistance states are due to interfacial contact resistance. To confirm this assumption, we investigate the contact resistance for the samples with different HfO_*x*_ thickness (see Supplementary Fig. 3 and Supplementary Note 2). Before measuring the contact resistance, the sample is intentionally broken down to minimize the possible CF contribution. A linear relationship between the resistance of broken-down sample and the HfO_*x*_ thickness with the intercept of 9.7 Ω is observed (see Supplementary Fig. 3a), which indicates that the contact resistance almost keeps constant at ~10 Ω for the sample with different HfO_*x*_ thickness. On the other hand, the temperature dependence of the contact resistance presents a metallic behaviour (see Supplementary Fig. 3b). Therefore, for *R*=8.4 kΩ case, where the contribution of contact resistance is much smaller than that for *R*=78 Ω, the decreasing tendency of *R* with increasing temperature reveals the intrinsic electrical transport mechanism in CF. Because of the same electrical transport mechanism resulted from *S* versus *T*, we believe the metallic increasing tendency of *R* with increasing temperature for *R*=78 Ω is due to the contribution of contact resistance, which results in the metallic behaviour as mentioned above. It should be mentioned that although the resistance of CF is still larger than the contact resistance for *R*=78 Ω, the resistance change of contact is larger than that for CF when *T* increases from 150 to 300 K. Therefore, the temperature-dependent behaviour of the contact resistance dominates the total measured resistance behaviour when *R*=78 Ω, leading to a metallic behaviour as shown in Fig. 2b. As we discussed below, the decreasing tendency of *R* with increasing temperature is the typical semiconductor transport behaviour, which can be understood by the small-polaron hopping transport model.

### Seebeck coefficient and resistance below 150 K

**Figure 3:**
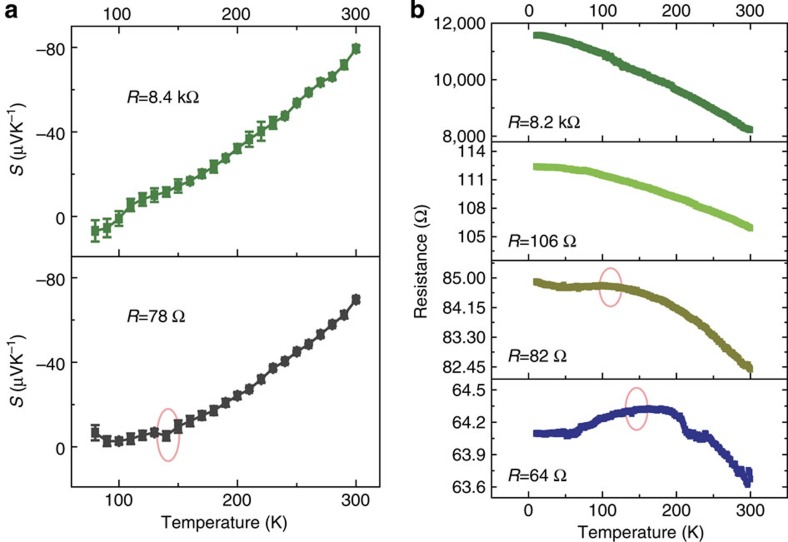
Measured SMT around 150 K. (**a**) Representative temperature dependence of the Seebeck coefficient when *R*=78 Ω and 8.4 kΩ between 78 and 300 K. (**b**) Temperature dependence of resistance from 10 to 300 K for several representative resistance states. The contact resistance has been deducted. The red circles represent the transition temperature regions.

Another remarkable result is the temperature dependences of *S* for *R*=8.4 kΩ and *R*=78 Ω are quite different at lower-temperature region below 150 K. As shown in Fig. 3a, in contrary to a continuous decrease tendency of the 8.4 kΩ, *S* of 78 Ω tends to be saturated when *T*<150 K. We attribute the saturated *S* at lower temperature to the possible SMT. First, in our case, the constant value of *S* about several μV K^−1^ below 150 K, which does not depend on the temperature anymore, is the typical metallic electrical transport behaviours^14^. Second, to further identify temperature-induced SMT, we measured temperature dependence of *R* for different resistance states from 64 Ω to 8.4 kΩ under the temperature from 10 to 300 K, as shown in Fig. 3b. For clarity, the contact resistance has been subtracted from every resistance value (see Supplementary Fig. 3b). For LRS of 82 and 64 Ω, two distinct regions with the opposite temperature dependences of *R* are observed, and the transition temperature between two regions decreases with increasing *R*. For *R*>106 Ω, the transition temperature becomes close to 0 K, and no clear SMT is observed. We speculate the observed SMT may be due to the temperature-induced Fermi level movement^22^, and more details need to be further clarified by theory.

## Discussion

We will show below the temperature dependence of *S* can be quantitatively explained by the small-polaron hopping transport model. Based on this model, the Seebeck coefficient now can be simplified to the sum of two terms^23^ (see Supplementary Note 3)


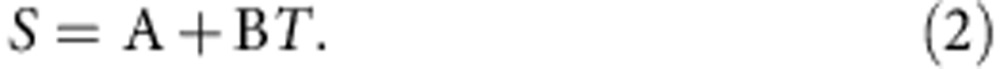


**Figure 4:**
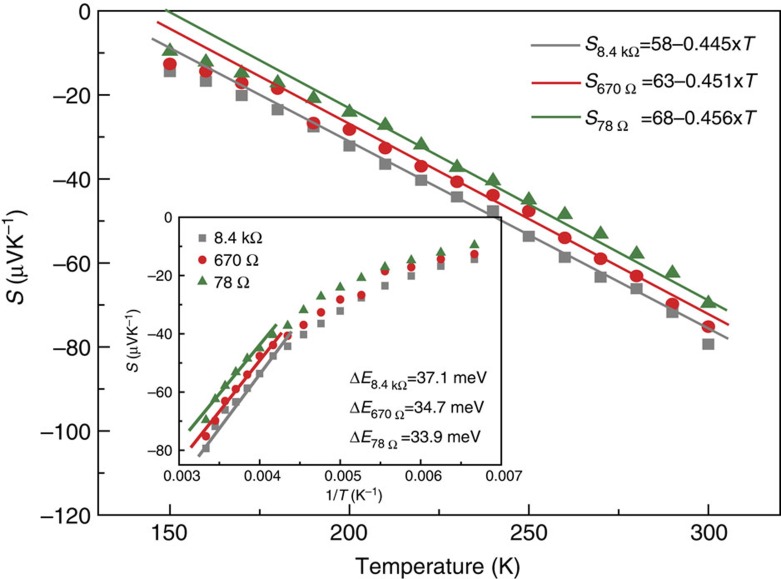
Fitting results of temperature-dependent Seebeck coefficient. The fitting results of the Seebeck coefficient *S* versus *T* for several representative resistance states (*R*=78 Ω, 670 Ω and 8.4 kΩ). The inset plots *S* versus *1/T* and the corresponding fitting results.

The first term *A* is the standard term, which is proportional to the average change of the entropy. The second term 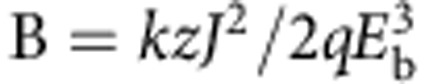 is proportional to the transported average lattice vibrational energy associated with charge hopping, where *k* is Boltzmann constant, *J* is the intersite transfer energy, *E*_b_ is the small-polaron binding energy and *z* is the number of nearest neighbours. Figure 4 shows the fitting results of Fig. 2a by equation (2) using parameters *A*=58, 63, 68 and *B*=−0.445, −0.451, −0.456 for *R*=8.4 kΩ, 670 Ω, 78 Ω, respectively. With decreasing the resistance, which is corresponding to the increasing of the oxygen vacancy concentration, the entropy contribution *A* increases. This occurs because of the addition of the active oxygen vacancy due to the increasing fraction of the filled unfavourable oxygen vacancy sites in energy as the small-polaron electron band broadens. Therefore, the average energy disparity between oxygen vacancy sites increases with the addition of oxygen vacancy. Otherwise, with the increasing of oxygen vacancy, the mean energy separation between oxygen vacancy decreases; as a result, the characteristic transfer energy for the intervacancy hopping increases, and thus the resultant *B* increases with oxygen vacancy concentration. In addition, by fitting *S* versus 1/*T* using the more general Mott Seebeck model^24^, the activation energy of electron hopping between oxygen vacancies Δ*E*=37.1, 34.7 and 33.9 meV for *R*=8.4 kΩ, 670 Ω and 78 Ω, respectively (see the inset of Fig. 4).

**Figure 5:**
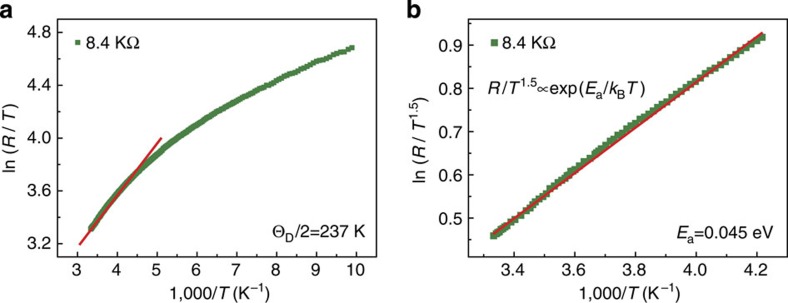
Fitting results of temperature-dependent resistance. (**a**) Inverse temperature dependence of ln(*R/T*) in high-temperature range. The temperature where the slope changes from linearity is *θ*_D_/2. (**b**) The fitting results of ln(*R/T*^1.5^) versus (1,000/*T*) above *θ*_D_/2 (237 K) by using small polaron-hopping model. The slope gives the activation energy from electrical measurement.

Furthermore, to minimize the effect of contact resistance, we take *R*=8.4 kΩ as an example to extract the charge transport parameters by using the small-polaron hopping model. First, we evaluate the Debye temperature by fitting the measured resistance in high-temperature range. Figure 5a shows the fitting results by plotting ln(*R*/*T*) versus 1/*T*. From this plot, the half Debye temperature, *θ*_D_/2=237 K, where the slope changes from linearity is determined. Second, according to the small-polaron hopping model (non-adiabatic)^25^, the expression for resistance takes the form


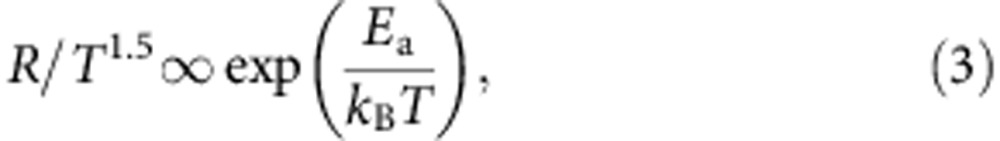


where *E*_a_ is the activation energy. Therefore, the activation energy can be further obtained by fitting the measured resistance using equation (3). The corresponding fitting results by using *E*_a_=0.045 eV is shown in Fig. 5b. The difference of the activation energy between here and the one obtained from fitting the Seebeck coefficient is due to the polaron-hopping energy^26^,^27^. Based on the value extracted here, the evaluated value for small-polaron coupling *γ*_p_=*E*_a_/*k*_B_*θ*_D_≈1.1. From the value of *γ*_p_, an estimation of the polaron effective mass, *m*_p_, can be obtained by using *m*_p_=m*exp(*γ*_p_) with m* denoting the rigid lattice effective mass. The calculated values of *γ*_p_ are found to be smaller than 4, indicating the weak electron–phonon interaction here^28^. These results further confirm that the charge transport is dominated by the hopping of small-polaron between vacancies.

Finally, by combining the electrical and the Seebeck measurement results, the carrier concentration of ~10^19^ cm^−3^ is estimated for *R*=8.4 kΩ (see Methods), which is corresponding to the oxygen vacancy density within the CF. More remarkably, although the carrier concentration in lower resistance state cannot be accurately estimated due to the involvement of contact resistances, the relative carrier concentration could be approximately estimated from the overall *R*-values. The carrier concentration for *R*=78 Ω will be ~107 times higher than that in *R*=8.4 kΩ, and the carrier concentration (~10^21^ cm^−3^) is still much less than that in epitaxial HfO_*x*_^29^. These results indicate that the oxygen vacancy density is still below the highest defect concentration and the electron transport in CF is dominated by hopping process.

In summary, we have measured the thermoelectric Seebeck effect in metal oxide-based RRAM and investigated the fundamental electronic transport properties of formed CF in RS process. We show, regardless of the resistance of RRAM, the charge transport in formed CF can be qualitatively described within the model of small-polaron hopping between oxygen vacancy sites. Moreover, a temperature-dependent SMT is observed for LRS of *R*<106 Ω with the transition temperature decreasing with increasing *R*. The thermoelectric Seebeck measurement, which promises to eliminate the possible contact contribution, opens a new avenue in investigating the electronic transport in RRAM-like devices.

## Methods

### Device fabrication

The RRAM devices with the dimension of 15 × 15 μm were obtained from Pt 30/Ti 5/SiO_2_ 70/W 40/Ti 10/HfO_*x*_ 8/Pt 60/Ti 5/SiO_2_ 70/Pt 30/Ti 5/SiO_2_/Si (nm) structures by standard nanofabrication process. Two side Pt 30/Ti 5 (nm) layers separated by 70 nm SiO_2_ layers from W 40/Ti 10/HfO_*x*_ 8/Pt 60/Ti 5 (nm) are used to heat the top and bottom surfaces of RRAM. The HfO_*x*_ layer was deposited by atomic layer deposition and the other layers were deposited by sputtering. The schematic structure of the sample is shown in Fig. 1a, and other SiO_2_ filling layers were also deposited around the device in nanofabrication process. The top and bottom heating layers out of the RRAM region were patterned with the width sharply increasing from 15 up to 200 μm to minimize the corresponding resistance contribution when evaluating temperature gradient.

### Seebeck effect measurements

Before the Seebeck effect measurement, the different resistance states were achieved by ingeniously controlling the *I*_CC_ in set process or the applied voltage in reset process, respectively. The operation voltage was supplied by a Keithley 2440 sourcemeter. Next, another two Keithley 2440 sourcemeters were used to provide the Joule heating current *I*_b_ and *I*_t_, and an Agilent 34411A voltmeter was used to measure the Seebeck voltage. The temperature dependence of the Seebeck coefficient was performed in a cryogenic probe station (Lakeshore, CRX-4K) with the temperature range of 78–400 K. The Seebeck voltage was also measured by sweeping *I*_t_ at each temperature point after the temperature was stabilized. The corresponding *R* versus *T*, especially below 78 K, was also measured by physical property measurement system (Quantum Design).

### Calibration of temperature gradient across HfO_*x*_ layer

We first measured the resistances of top (*R*_t_) and bottom (*R*_b_) heating layer as a function of temperature, and found both the resistances of top and bottom heating layer could be well fitted by a linear function. As an example, we present the typical *R*_t_ versus *T* and the corresponding linear fitting results (see Supplementary Fig. 2a). By using the linear fitting results, we then could determine the temperature difference between top and bottom surfaces of our sample. We measured *R*_t_ as a function of top heating current (*I*_t_), and found that *R*_t_ versus *I*_t_^2^ could be well fitted by a linear function (see Supplementary Fig. 2b). The well linear fitting results indicate the top surface temperature (*T*_t_) is proportional to *I*_t_^2^. Similarly, by simultaneously measuring *R*_b_ when sweeping *I*_t_, we could also determine the linear dependence of bottom surface temperature (*T*_b_) on *I*_t_^2^. Therefore, the temperature difference between top and bottom surface (*T*_t_*–T*_b_) as a function of *I*_t_^2^ were obtained. For simplicity, we ignore the temperature difference across metal layers, because the thermal conductivities of these layers are much larger than those for insulating SiO_2_ and HfO_*x*_ layers. Therefore, the temperature difference across HfO_*x*_ layer 
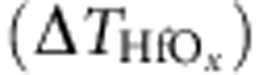
 and each SiO_2_ layer 
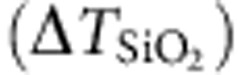
 can be given by


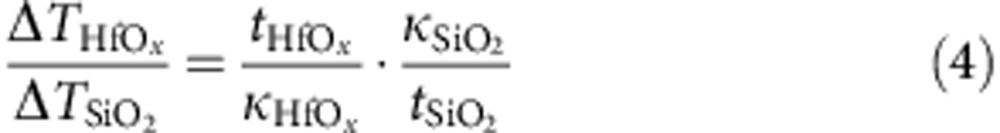


and


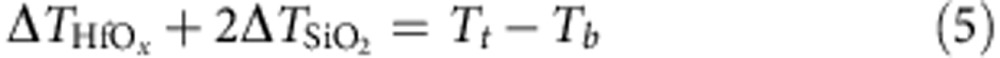


where 
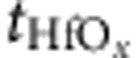
, 
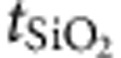
is respectively the thickness of HfO_*x*_ and SiO_2_ layer, and 
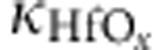
, 
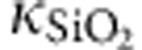
 is respectively the thermal conductivity of HfO_*x*_ and SiO_2_ layer. As the relative thermal conductivity change between the HfO_*x*_ and SiO_2_ layers is <5% within the range of 80–300 K^30^,^31^, we use the relative thermal conductivity ratio (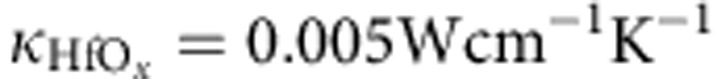 and 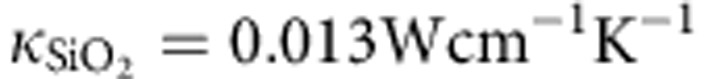) at 300 K to calculate the temperature distribution in our sample. According to equations (4) and (5), we can get the temperature difference across HfO_*x*_ layer, which shows a linear dependence on *I*_t_^2^, and is insensitive with ambient temperature (see Supplementary Fig. 2c).

### Calculation of oxygen vacancy concentration

According to the activation energy equation proposed by Austin and Mott^28^, *E*_a_=*W*_H_+*W*_D_/2, when *T*>*θ*_D_/2, where *W*_H_=*E*_a_−Δ*E* is the polaron-hopping energy^27^, *W*_D_ is the disorder energy. By using *E*_a_=0.045 eV and Δ*E*=0.037 eV, we can get *W*_H_=0.008 eV and *W*_D_=0.074 eV. Based on the Millar–Abraham theory^32^, *W*_D_=0.3*e*^2^/*ε*_s_*R*_O_, where *ε*_s_ is the static dielectric constant and *R*_O_ is the average spacing between oxygen vacancies. If we chose *ε*_S_=21 for HfO_*x*_ film^33^, the *R*_O_ will be 3.5 nm, and the oxygen vacancy concentration *n* is ~10^19^ cm^−3^ for *R*=8.4 kΩ. Furthermore, we can estimate *n*~10^21^ cm^−3^ for *R*=78 Ω. For both cases, the oxygen vacancy concentration is still within the range of hopping transport model (below the critical value of 10^22^ cm^−3^).

## Author contributions

M.W., C.B., L.L. and M.L. designed this work; M.W.and C.B. designed and fabricated the devices, and carried out the Seebeck effect measurement; M.W. preformed the temperature-dependent resistance measurement; L.L. interpreted the experiment results; all authors discussed the experiments and contributed to the manuscript preparation. M.L. coordinated and supervised the whole work.

## Additional information

**How to cite this article:** Wang, M. *et al*. Thermoelectric Seebeck effect in oxide-based resistive switching memory. *Nat. Commun.* 5:4598 doi: 10.1038/ncomms5598 (2014).

## Supplementary Material

Supplementary Information Supplementary Figures 1-3, Supplementary Notes 1-3 and Supplementary Reference
