# Supplementary material for: Thermoelectric Seebeck effect in oxide-based resistive switching memory
Source: Nat Commun. 2014 Aug 20;5:4598. doi: 10.1038/ncomms5598 (PMC4143917; doi:10.1038/ncomms5598)
Supplement: Supplementary Information — Supplementary Figures 1-3, Supplementary Notes 1-3 and Supplementary Reference [file ncomms5598-s1.pdf]

SUPPLEMENTARY FIGURES

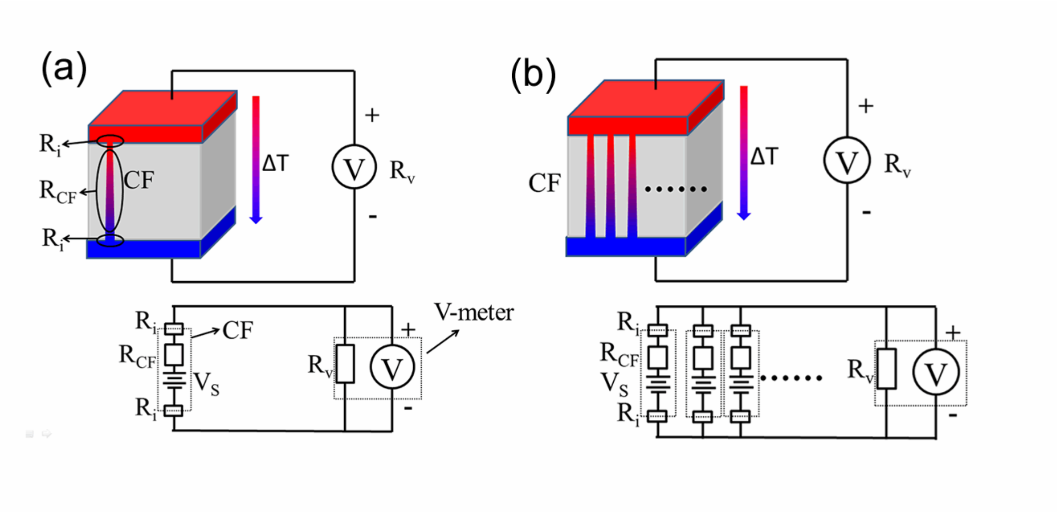

Supplementary Figure 1. Schematic illustration of Seebeck measurement in RRAM cell. **(a)** The equivalent circuits for the single conductive filament (CF) RRAM case. **(b)** The equivalent circuits for multiple CFs RRAM case.

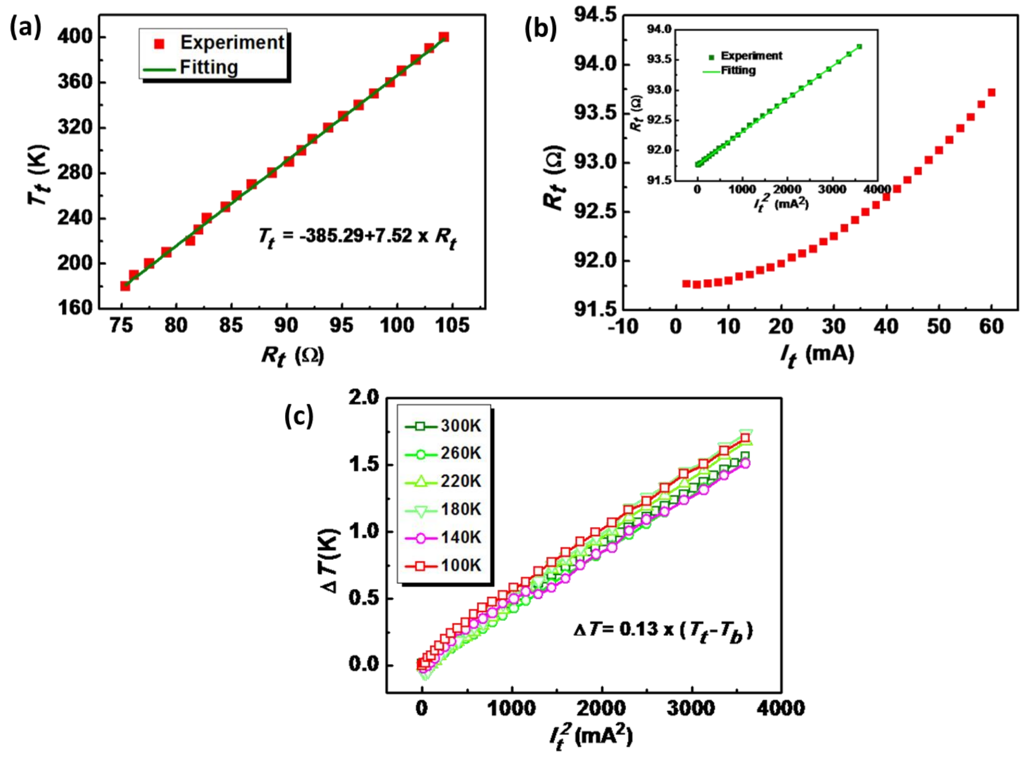

Supplementary Figure 2. Calibration of temperature difference across HfO_x layer. (a) The measured top heating layer resistance R_t (symbols) as a function of temperature and the corresponding linear fitting results (solid line). **(b)** The measured top heating layer resistance R_t as a function of top heating current I_t. The inset shows the corresponding R_t versus I_t^2 and linear fitting results. **(c)** Temperature difference across HfO_x layer (ΔT_{HfO_x}) as a function of I_t^2 at different temperatures.

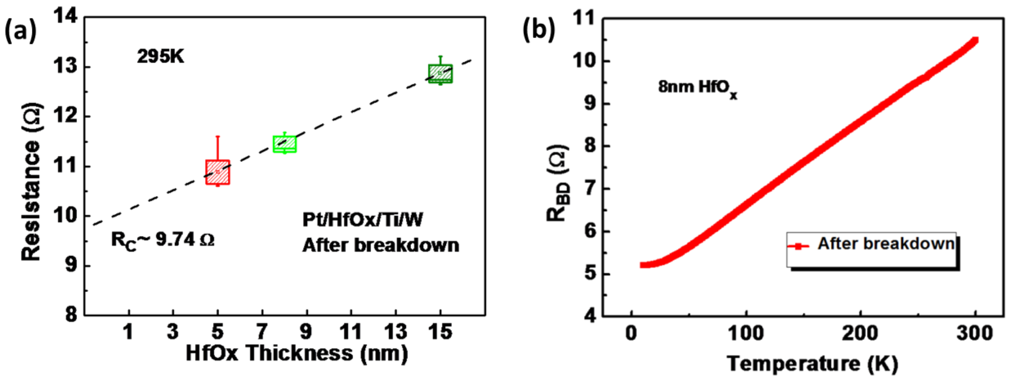

Supplementary Figure 3. Estimation of contact resistance. (a) The measured resistance of RRAM after breakdown (R_{BD}) for the three samples with 5 nm, 8 nm, and 15 nm HfO_x layer at 295 K. The intercept is the value of contact resistance (9.7 Ω). **(b)** R_{BD} as a function of temperature from 10 K to 300 K for 8 nm-thickness RRAM device.

SUPPLEMENTARY NOTES

Supplementary Note 1: Thermoelectric Seebeck measurement in RRAM

Seebeck voltage is created by a temperature gradient, and thus the Seebeck device can be simplified to be a voltage-source equivalent circuit. For the single conductive filament (CF) RRAM case, the voltage-source equivalent circuit is shown in the bottom of Supplementary Fig. 1a. The top panel of Supplementary Fig. 1a shows the measurement schematic of the RRAM device with the single CF, where R_{CF} is the internal resistance of CF, R_i is the interfacial contact resistance, and R_V is the internal resistance of voltmeter. The thermoelectric Seebeck voltage (V_S, see the bottom panel of Supplementary Fig. 1a) created by temperature gradient can be written as:

$$V_S = 2S_i \Delta T_i d_i + S_{CF} \Delta T_{CF} d_{CF} \tag{1}$$

where S_i is the interfacial Seebeck coefficient of the CF/electrode (top or bottom) interface, ΔT_i is the temperature gradient across the CF/electrode interface, d_i is the effective thickness of interface contact, S_{CF} is the Seebeck coefficient of CF, ΔT_{CF} is the temperature gradient along CF, d_{CF} is the length of CF. Therefore, the measured voltage by voltmeter should be:

$$V_{Measured} = IR_V = \frac{V_S}{2R_i + R_{CF} + R_V} R_V \tag{2}$$

where I is the current driven by V_S in the closed equivalent circuit. In real measurement, since $R_V > 10$ GΩ >> $R_i + R_{CF}$, Eq. (I.2) becomes $V_{Measured} \approx V_S$. On the other hand, by considering $d_{CF} >> d_i$, Eq. (1) can be further written as $V_S \approx S_{CF} \Delta T_{CF} d_{CF}$. Therefore, the measured Seebeck voltage $V_{Measured} \approx V_S = S_{CF} \Delta T_{CF} d_{CF}$ will not depend on the interfacial contact.

For the device with two or more CFs (see Supplementary Fig. 1b), Eq. (2) becomes

$$V_{Measured} = IR_V = \frac{V_S}{(2R_i + R_{CF})/n + R_V} R_V \quad (3)$$

where n is the number of CFs. In this case, we can still obtain $V_{Measured} \approx V_S$, because $R_V >> 2R_i + R_{CF}$. Hence, the measured voltage will also not depend on the number of CFs.

Supplementary Note 2: Determination of contact resistance

To determine contact resistance (R_C) in our electrical measurement, we fabricated the other two control RRAM samples with the same RRAM multilayer structures except the thickness of HfO_x layer t_{HfO_x} was 5 nm and 15 nm, respectively. For simply, no heating layers were deposited, and only the top electrodes of RRAM were patterned into 300×300 μm^2 dots for measurement. If we assume R_C does not depend on the resistance states of RRAM, the measured resistance of RRAM after breakdown (R_{BD}) will be mainly due to contact resistances. The measured R_{BD} for the three samples with 5 nm, 8 nm, and 15 nm HfO_x layer at 295 K are shown in Supplementary Fig. 3a. R_{BD} versus t_{HfO_x} can be linearly fitted with the intercept of 9.7 Ω, which indicates R_{BD} approaches to a constant resistance value when HfO_x thickness is near to 0 nm. Therefore, it is reasonable to take 9.7 Ω as the contract resistance in our samples. For t_{HfO_x} =8 nm, R_{BD} is about 11.4 Ω, thus internal resistance of RRAM structure is about 1.7 Ω, which is much smaller than the interfacial contact resistance. The much smaller internal resistance further confirms R_{BD} for 8 nm HfO_x sample is mainly from contact resistance. Supplementary Fig. 3b shows R_{BD} as a function of temperature from 10 K to 300 K. It can be seen that R_{BD} shows a metallic increase tendency with increasing temperature. The resistance change is about 5.2 Ω from 10 K to 300 K, which has the same magnitude as the corresponding resistance change for R=78 Ω in Fig. 2b. These results clearly demonstrate that the metal-like resistance behaviors for low resistance states with R < 82 Ω is due to contact resistance. R_{BD} shown in Supplementary Fig. 3b, which is mainly from R_c, is also

used to deduct contact resistance contribution for *R*-*T* measurement (Fig. 3b).

Supplementary Note 3: Seebeck coefficient calculation based on small-polaron hopping transport

Our calculation is based on the work of D. Emin [1], where the Seebeck coefficient S_{ij} between two-sites is found to be

$$S_{ij} = -\frac{1}{qT}[\frac{\varepsilon_i + \varepsilon_j}{2} + E_T^{ij} - \zeta] \quad (4)$$

ε_i and ε_j are respectively the equilibrium electron energy levels, and ζ is the chemical potential of the electrons. The interaction energy between electron and lattice is carried by the term E_T^{ij}. In particular, the vibrational energy transfer from an initial state *i* to a finial state *j* is given by

$$E_T^{ij} = \frac{1}{2}(\varepsilon_j - \varepsilon_i)(\Gamma_i - \Gamma_j)/(\Gamma_i + \Gamma_j) \quad (5)$$

Γ_i and Γ_j denote the electron-lattice coupling strengths associated with the occupation of the two respective sites. For sufficiently small energy barrier between these two states, $(\Gamma_i - \Gamma_j) \propto (\varepsilon_j - \varepsilon_i)$. Then Eq. (5) is written as

$$E_T^{ij} = C(\varepsilon_j - \varepsilon_i)^2 \quad (6)$$

Averaging of this term yields a contribution to the Seebeck coefficient which increases linearly with temperature: *BT*, where $B = 8kCT/q$, $C = zJ^2/16E_b^3$ with *J* the intersite transfer energy, E_b the small-polaron binding energy, and *z* the number of nearest neighbors. In contrast, the part $\left(\frac{\varepsilon_i + \varepsilon_j}{2} - \zeta\right)$ of Eq. (4) is written as *TΔS* with *ΔS* denoting the average change of the system entropy when a carrier is injected into it. Therefore, Seebeck coefficient is reasonably close to the observed form, $S_{ij} = A + BT$, with $A = \Delta S$ and $B = 8kCT/q$.

SUPPLEMENTARY REFERENCES

[1] Emin, D. Thermoelectric power due to electronic hopping motion. *Phys. Rev. Lett.* **35,** 882-885 (1975).
